# Pulmonary Tuberculosis Incidence and Risk Factors in Rural Areas of China: A Cohort Study

**DOI:** 10.1371/journal.pone.0058171

**Published:** 2013-03-12

**Authors:** Wei Chen, Wen Shu, Min Wang, Yongchun Hou, Yinyin Xia, Weiguo Xu, Liqiong Bai, Shaofa Nie, Shiming Cheng, Yihua Xu

**Affiliations:** 1 Department of Epidemiology and Biostatistics, School of Public Health, Tongji Medical College, Huazhong University of Science and Technology, Wuhan, China; 2 National Center for Tuberculosis Control and Prevention, Chinese Center for Disease Control and Prevention, Beijing, China; 3 Medical Reform Office, Wuhan Health Bureau, Wuhan, China; 4 Jiangsu Provincial Center for Disease Control and Prevention, Nanjing, China; 5 TB Institute for Tuberculosis Control and Prevention of Hunan Province, Changsha, China; Universidade Federal do Acre (Federal University of Acre), Brazil

## Abstract

The incidence of tuberculosis (TB) and its risk factors in China remains unclear. This study examined TB incidence and relative risk factors in rural areas of China. Participants (n = 177,529) were recruited in Xiangtan County (in the central area of China) and in Danyang County (in the eastern area of China) in 2009 and a followed-up study was conducted for one year. The incidence density of pulmonary TB and smear-positive TB were 91.6 (95% CI: 78.7, 106.0) per 100,000 person-year and 36.7 (95% CI: 33.1, 52.4) per 100,000 person-year respectively in Xiangtan, and 47.3 (95% CI: 38.2, 57.5) per 100,000 person-year and 22.7 (95% CI: 16.5, 30.8) per 100,000 person-year in Danyang. The medical history of TB was associated with TB, with the relative risk (RR) of 7.00 (95% CI: 2.76, 17.18) in Xiangtan and that of 31.08 (95% CI: 13.22, 73.10) in Danyang. The association between TB and per capita living space over median was found in Xiangtan, with the RR of 1.86 (95% CI: 1.15, 3.01). No association was found between TB and the insurance status, the contact history with TB, the history of diabetes, smoking, or per capita annual income. The host genetic susceptibility, and social factors such as education and income could be considered in future studies.

## Introduction

In 2010 an estimated 8.8 million (95% Confidence interval: 8.5, 9.2 million) new tuberculosis (TB) cases occurred in the world, equivalent to 128 cases per 100,000 population [1]. China had ∼1,000,000 TB cases, the second largest number in the world, just behind India. In China, about 80% of TB patients live in rural areas [2]. In China’s 2010 National TB Prevalence Survey, the prevalence of active pulmonary TB in rural areas was about 1.8 times that of urban areas; that of sputum smear-positive TB was about 1.6 times that of urban areas. This large population of TB patients has a high prevalence of anti-TB drug resistance, which increases the rate of treatment failure and costs of control, and is a major challenge to public health for China [3].

TB incidence–one of the most important surveillance indicators–reflects the impact of TB on public health. Since 1979, China has conducted five national TB prevalence surveys. The infection and prevalence of TB were investigated; however, little data on TB incidence were reported. While a majority of researchers have paid close attention to prevalence, only a few have focused on TB incidence in China [4]–[6]. One important reason is that to investigate TB incidence, the particular population needs to be sampled and followed up for an appropriate period of time to collect data about new cases and related exposure–a time-consuming process, requiring many human and financial resources. The course of the disease is uncertain and not easy to follow, making it very difficult to estimate TB incidence based on TB prevalence.

Besides incidence, the following risk factors relative to TB occurrence are also unclear in rural populations in China: being male, poor living conditions, smoking, history of TB, and contact with TB patients [7]–[9]. References on TB incidence- influencing factors from China are seldom cited in the literature.

We could not speculate about TB incidence using the new TB notifying rate directly in most developing countries, because of patients’ incomplete registration forms, so we did not have access to fully accurate TB incidence data or were unable to identify the influencing factors. We conducted a population-based cohort study in two counties of Eastern and Middle China to get TB incidence rates in rural areas and to analyze the association between risk factors and TB.

## Materials and Methods

### Study Design

Our study included a baseline study, a one-year follow-up, and an endpoint survey. In our baseline survey, trained staff administered a standard questionnaire to obtain baseline information which included demographic data, medical history of TB symptoms or related chronic diseases, and lifestyle risk factors. We conducted household interviews regarding TB symptoms of all the residents in the study sites. Individuals who had suspect TB symptoms and/or history of TB were encouraged to obtain chest X-rays and collect three sputum specimens for smear and culture tests. According to the TB diagnosis standard (national criterion No. WS288-2008), people who did not have TB were eligible for the follow-up cohort observation.

The cohort was followed up for one year and TB incidence information was collected. During the observation period, new TB cases were detected through the following procedures during the follow-up period: (1) Routine detection: we carried out a media campaign and health education regarding TB to enhance public awareness of TB prevention and then actively mobilized the follow-up population who had suspect TB symptoms to designated TB health facilities; (2) by strengthening follow-up screening for high-risk groups such as those who had close contacts with smear-positive patients; (3) by detecting new TB cases through household clue investigations, community recommendations, and health examinations.

At the endpoint of the follow-up, all existing populations received symptom interviews with the same investigation method as the baseline survey.

### Study Site Sampling

We used a convenience sampling method to choose selected provinces; economic conditions were important factors for choosing study fields. We selected Danyang County of Jiangsu Province in Eastern China and Xiangtan County of Hunan Province in Middle China as representatives of good and poor financial status in the rural areas, respectively.

Danyang is a county in south Jiangsu Province, with a population of 0.81 million and an area of 1,047 km^2^. This county ranks 18th of the 100 richest counties in China. During 2006–2008, the average notification rate of active pulmonary TB was 59.74/100,000; that of new sputum smear-positive TB was 33.03/100,000. Another study site was Xiangtan County, in the mid-eastern part of Hunan Province, with a population of 1.09 million and an area of 2,372 km^2^. The economic status of Hunan is not as good as that in Jiangsu. The average net income of rural inhabitants in Xiangtan County was 6,458 RMB (official currency of China) in 2009, compared with 10,058 RMB in Danyang in the same period. During 2006–2008, the average notification rate of active pulmonary TB in Xiangtan County was 101.10/100,000; that of new sputum smear-positive TB was 63.52/100,000.

The sampling process was based on the average notification rate of new smear-positive TB during 2006–2008. Townships in the two counties were stratified into three levels (low, middle, and high level) based on their TB notification rate. One township was randomly selected from each level. We selected two towns from Danyang County and three from Xiangtan County.

All local residents (excluding people absent for more than six months during that year) and permanent residents immigrating from other areas/provinces for more than three months were included in the survey.

### Initial Assessment for Exposed Variables

Gender, age, marital status, occupation, and educational level were considered as background factors. These factors were demographic characteristics which would not change by public health intervention. Insurance status, history of TB, history of contact with TB patients, history of diabetes, smoking (former or current), per capita annual income, and per capita living space were classified as potential risk factors.

If a person had access to any type of medical insurance, we defined the insurance status as exposed. A history of TB was determined by medical records as diagnosed and registered by TB dispensaries. History of contact with TB patients was based on self-reported information from the questionnaire. A history of diabetes was identified by a positive response from participants to the question, “Has a doctor ever told you that you have diabetes?” Cigarette smoking was defined as having smoked at least 100 cigarettes in one's lifetime. For analysis, per capita annual income and per capita living space were divided into “below median” and “over median.”

### Measurement of Outcome

Laboratory-confirmed TB cases were defined as sputum smear-positive (including scanty positive) or sputum culture-positive for *mycobacterium tuberculosis* (*M. tuberculosis*). Sputum smear-negative and culture-negative TB were defined as a clinical illness, also chest X-rays consistent with active TB which did not respond to broad-spectrum antibiotics but did respond to anti-TB treatment. Participants were classified as lost-to-follow-up if they did not attend for review (scheduled or unscheduled) during the study period and could not be traced; their records were deleted from analysis.

### Statistical Approach

We performed all statistical analyses using SPSS for Windows (version 12.0). Continuous variables such as age, per capita annual income, and per capita living space were divided into categorical variables. We used the following calculation of incidence density. The all detected new TB cases during follow-up and endpoint periods was the numerator; person-time based on length of the person’s exposed/unexposed time was the denominator. To compare the incidence density between Xiangtan County and Danyang County, the incidence was standardized by age and gender. A Poisson regression model was fitted to identify risk factors associated with new TB cases. Insurance status, history of TB, history of diabetes, smoking (former or current), per capita annual income, and per capita living space were included as factors in the model.

Each model was then repeated with an adjustment approach for the following potential confounders: gender, age, marital status, education, and occupation. *P* value less than 0.05 was considered statistically significant, with a 95% confidence interval (95% CI) to determine adjusted relative risk.

### Ethical Review

The study protocol was approved by the institutional review boards of the Tongji Medical College, Huazhong University of Science and Technology. We obtained informed written consent from all participants or the next of kin, carers or guardians on the behalf of the minors/children participants after the study protocol had been explained to them.

## Results

### Demographic Characteristics of Participants and Patients

The eligible population in our cohort study was 192,166; after the one-year follow-up, 177,529(92.4%) completed the study: 84,526 participants in Xiangtan County and 93,003 in Danyang County, including 90,574 men and 86,955 women (Table 1). Over one quarter of the population was over 55 years of age in both counties. The main occupations were factory worker, rural laborer, or peasant. The population in Danyang had higher educational levels than those in Xiangtan.

**Table 1:** Demographic Characteristics of Participants in the Study, 2009–2011.

Variable	XiangtanN = 84526		DanyangN = 93003		TotalN = 177529	
	Participants	ActiveTB case	Participants	ActiveTB case	Participants	Active TB case
Gender						
male	44262	58	46312	32	90573	90
Female	40264	14	46691	13	86955	27
Age(years)						
0–14	11021	0	5832	0	16853	0
15–59	58986	39	66291	24	125277	63
≥60	14519	33	20880	21	35399	54
Marital status						
single	26279	5	18978	1	45257	6
married	53829	62	66652	34	120481	96
divorced/widowed	4400	5	7320	10	11720	15
Occupation						
factory worker, rural laborer, peasant	57020	66	58231	26	115251	92
Retired, household and unemployed	9212	5	14858	14	24070	19
other	18290	1	19599	5	37849	6
Educational level						
illiteracy	8334	9	8189	3	16523	12
primary school	27145	34	25122	18	52267	52
junior school	37370	27	45436	21	82806	48
senior school or higher	11578	2	14185	3	25763	5

In the period under observation, 117 new TB cases occurred: 72 cases in Xiangtan County, 45 in Danyang County. The sex ratio was 4.1∶1 in Xiangtan and 2.5∶1 in Jiangsu. None of the patients were under 15 years of age, in both fields. The factory worker, rural laborer, peasant group had the most patients in the three occupational groups. Primary school and junior school education levels accounted for 85% of the patients.

### Crude and Standardized Incidence Density in Two Counties

We observed 72 new TB cases in Xiangtan County during the follow-up period, including 29 sputum smear-positive cases. The crude incidence density of active TB was 85.3 (95% CI: 66.7, 107.4) per 100,000 person-year in total, and that of sputum smear-positive TB was 34.3 (95% CI: 23.0, 49.3) per 100,000 person-year (Table 2). When it was standardized by merged population, the gender-specific incidence density of both active TB and sputum smear-positive TB increased. The incidence was significantly higher among men than women whether before or after standardization (*P*<0.001).

**Table 2:** Incidence of Active TB and Sputum Smear-Positive TB in the Study, 2009–2011.

Variable	Person-year	No.	Crude		Standardized^a^	
			Incidence density^b^	95% CI	Incidence density^b^	95% CI
**Active TB**						
**Xiangtan**						
male	44203.1	58	131.2	99.6,169.6	144.3	120.8,171.0
Female	40245.9	14	34.8	19.0, 58.4	36.7	26.8,50.5
total	84449.0	72	85.3	66.7,107.4	91.6	78.7,106.0
**Danyang**						
male	44166.0	32	72.5	49.6,102.3	67.4	51.8,86.1
female	44490.2	13	29.2	15.6,50.0	26.3	18.2,37.9
total	88656.2	45	50.8	37.0,68.0	47.3	38.2,57.5
**Sputum smear-positive TB**						
**Xiangtan**						
male	44203.1	22	49.8	31.2,75.4	55.0	41.0,72.0
Female	40245.9	7	17.4	7.0,35.8	17.7	9.9,27.7
total	84449.0	29	34.3	23.0,49.3	36.7	33.1,52.4
**Danyang**						
male	44166.0	16	36.2	20.7,58.8	32.6	22.0,45.8
female	44490.2	6	13.5	4.9,29.4	12.3	6.5,21.7
total	88656.2	22	24.8	15.6,37.6	22.7	16.5,30.8

Abbreviation: CI, confidence interval.

a Standardized by merged population distribution.

b per 100,000 person-year.

We detected 45 active cases in Danyang County, including 22 sputum smear-positive TB cases. The overall crude incidence density of active TB was 50.8 (95% CI: 37.0, 68.0) per 100,000 person-year in total; that of sputum smear-positive TB was 24.8 (95% CI: 15.6, 37.6) per 100,000 person-year. The number dropped to 47.3 (95% CI: 38.2, 57.5) per 100,000 person-year and to 22.7 (95% CI: 16.5, 30.8) per 100,000 person-year when standardized.

### Incidence Density in Exposed Variables

The highest incidence density appeared in the population with a medical history of TB in the two study sites (Table 3). In Xiangtan County the figure was 1,578.0 per 100,000 person-year; in Danyang County the number was 2,706.3 per 100,000 person-year. While the smoking group had the third highest incidence in Xiangtan, the diabetes group was in this rank in Danyang.

**Table 3:** Incidence and Relative Risk Under Exposed/Unexposed Conditions in the Study, 2009–2011.

Variable	No. of persons	Person -year	No. of active case	IncidenceDensity^a^	Relative risk	95%CI
Xiangtan						
Insurance status						
no	3068	3062.9	1	32.7	1	
yes	81422	81351.4	71	87.3	2.67	0.37,19.24
History of TB						
no	84076	84005.5	65	77.4	1	
yes	450	443.6	7	1578.0	20.39	9.35,44.47
Contact history with TB patients						
no	82959	82880.6	66	79.6	1	
yes	1426	1421.0	5	351.9	4.42	1.78,10.97
History of diabetes						
no	84109	84037.4	71	84.5	1	
yes	415	409.7	1	24.5	2.89	0.40,20.79
Smoking(former or current)						
no	65310	65258.6	45	69.0	1	
yes	19216	19190.4	27	140.7	2.04	1.27,3.29
Per capita annual income						
below median	41287	41208.1	36	87.4	1	
over median	43239	43147.3	36	83.4	0.96	0.60,1.52
Per capita living space areas						
below median	48610	48530.2	30	61.8	1	
over median	35916	35908.8	42	117.0	1.89	1.18,3.02
**Danyang**						
Insurance status						
No	1292	1236.2	0	0	-	
Yes	90543	86328.4	45	52.1	-	
History of TB						
no	92609	88286.7	35	39.6	1	
yes	394	369.5	10	2706.3	68.27	33.81,137.90
Contact history with TB patients						
no	91691	87413.8	41	46.9	1	
yes	1302	1232.8	4	324.5	6.92	2.48,19.31
History of diabetes						
no	92106	87805.0	42	47.8	1	
yes	897	851.3	3	352.4	7.37	2.28,23.77
Smoking(former or current)						
no	72338	68907.3	35	50.8	1	
yes	20665	19748.9	10	50.6	0.99	0.49,2.01
Per capita annual income						
below median	24870	23779.2	17	71.5	1	
over median	68131	64875.0	28	43.2	1.41	0.79,2.51
Per capita living space areas						
below median	39108	37445.8	18	4.8	1	
over median	53894	51209.5	27	52.7	1.10	0.60,1.99

a per 100,000 person-year.

In Xiangtan County, except for the group of per capita living space over median, people with characteristics such as access to insurance, or a history of diabetes had an incidence below 100 per 100,000 person-year. All the patients in Danyang County had insurance; the incidence changed from 4.8 to 71.5 per 100,000 person-year under different exposure conditions.

### Relative Risk of Exposed Variables

In univariate analysis, people who had a history of TB or TB contact history were more likely to progress to TB. In addition, smoking and per capita living space over median were significant risk factors in the Xiangtan population, while diabetes was significant in the Danyang population (Table 3).

The Poisson model (Table 4) also showed that people who had TB previously were more likely to develop TB again; the relative risk was 7.0 (95% CI: 2.76, 17.18) in Xiangtan and 31.1 (95% CI: 13.2, 73.1) in Danyang; however, the significance of contact history as a risk factor disappeared. In Xiangtan County, subjects in the group of per capita living space over median might be 1.8 times more likely (RR = 1.86, 95% CI: 1.15, 3.01) to develop TB. We also analyzed other risk factors (insurance status, history of diabetes, smoking, per capita annual income), but found that none of these factors had significant association with TB occurrence.

**Table 4:** Relative Risk of Exposed Factors Associated with TB in the Study, 2009–2011.

Variable	Xiangtan			Danyang		
	Relative risk^a^	95%CI	*P*	Relative risk^a^	95%CI	*P*
Access to insurance status	2.31	0.32,16.69	0.408	–	–	–
History of TB	7.00	2.76,17.78	<0.001	31.08	13.22,73.10	<0.001
Contact history with TB patients	2.40	0.88,6.53	0.086	1.49	0.46,4.86	0.510
History of diabetes	1.31	0.17,9.97	0.795	3.05	0.88,10.55	0.078
Smoking (former or current)	0.65	0.38,1.10	0.108	0.48	0.22,1.03	0.059
Per capita annual income over median	1.00	0.62,1.62	0.990	0.68	0.36,1.25	0.211
Per capita living space over median	1.86	1.15,3.01	0.010	1.07	0.59,1.96	0.822

Abbreviation: CI, confidence interval.

a Adjusted for all the background factors: gender, age, marital status, occupation and educational level.

## Discussion

In our cohort study, the incidence density of active TB ranged from 26.3 to 144.3 per 100,000 population; that of sputum smear-positive TB changed between 12.3 to 55.0 per 100,000 population in different gender and study sites. Xiangtan County suffered a more serious TB epidemic situation than Danyang County. Different economic levels may account for this. In the literature review, there were no related data about rural areas of China; however, in rural areas of Africa [10], the TB incidence rate of smear-positive pulmonary TB was 65 cases per 100,000 population; in 19 Pacific Island countries and territories, the rate was 52 cases per 100,000 population [11]; in Australia, 5.4 cases per 100,000 population [12]. In developing countries, especially in rural areas, TB incidence is much higher than that of cities, these differences among various countries could be due to different levels of economic development and the difference of financial input on TB diagnosis and treatment [13].

China has the largest agricultural population in the world: ∼674 million rural inhabitants. We estimate that if there are no effective interventions China will have at least 318,802 new TB cases in rural areas every year. China’s 2010 National TB Prevalence Survey also showed that the prevalence of active TB in rural areas was 569 per 100,000 population. Therefore, effective intervention strategies must be implemented to control TB in rural areas of China.

In our study, the TB incidence among men in Xiangtan and Danyang Counties was more than twice that of women. The distinction between public health risks of infection and different lifestyles may account for this gender difference. A recent study showed that concealment of a disease status such as TB happened more among females because of fear of losing social status, marital problems, or harmful reactions from the community [14].

Unlike other studies [15]–[17], we did not identify a history of contact with TB patients as an important risk factor associated with TB occurrence. In our study, investigation of contact history was based on self-report, so it was very difficult to avoid recall bias. In addition, there was no simple way to identify a TB patient in the population, so certainty of contact was weakened to some extent. But the higher incidence of TB in the population from contact with TB patients may still require more evidence to prove. Once *M. tuberculosis* infection has occurred, the risk for development of TB is influenced by several factors: duration of the infection; intrinsic characteristics such as age, body mass index, *Bacillus Calmette-Guerin*(BCG)immunization history, HIV infection, host genetic susceptibility, etc. So a higher incidence of TB may be caused by increased risk of exposure to *M. tuberculosis*.

Moreover, different contact types with intensity and duration, and differences in experiences of exposure to *M. tuberculosis* may result in incidence diversity of TB between male and female subjects. A study from a rural district in Malawi, Africa, suggested that identifiable recent contacts with known smear-positive cases accounted for 12.5% of the TB burden [18]. Therefore, programs such as contact investigations, which identify and preventively treat persons recently infected with *M. tuberculosis*, may have a substantial effect on controlling TB epidemics [19]. However, we could not assess the contact and exposure types of daily contacts in our subjects, but one could speculate that a considerable number of these contacts could have active TB and could have contributed to the continued spread of *M. tuberculosis* infection in their households and communities.

Another risk factor associated with TB is a history of TB, which has been documented by other research [20], [21]. Once a person is infected by *M. tuberculosis*, it persists for a long time in a process called latent TB infection (LTBI). LTBI has traditionally been considered to involve the TB bacilli remaining in a non-replicating state (dormant) in old lesions–but still retaining their ability to induce reactivation and to cause active TB when a disruption of the immune response occurs [22]. In a country with low-incidence of TB, comparison of the characteristics between relapse and re-infection subgroups revealed that only the presence of cavitary disease was associated with relapse [23]. Since TB recurrence is rarely due to re-infection, the TB recurrence was probably sustained during residence in a country with a high incidence of TB [24].

The prevalence of diabetes mellitus is increasing rapidly worldwide and has been reported to be significantly associated with increased risk of TB [25]–[27]. In 2008, 92.4 million adults in China were suffering from diabetes (50.2 million men and 42.2 million women) and 148.2 million adults (76.1 million men and 72.1 million women) were pre-diabetic [28]. Our study failed to indicate the association between diabetes mellitus and TB in the population. Research to examine the implications of these findings for TB control is urgently needed.

Smoking and alcohol use–two risk factors for TB mentioned in many studies [29]–[31] – were not quantitatively investigated in our study. We investigated smoking as a qualitative variable, but could provide no further information with regard to smoking duration and quantity. We did not consider alcohol use in the questions we investigated, so the association could not be evaluated.

TB research in other rural sites in China showed similar results of negative correlation of TB with socioeconomic status [2]. In one other study [32], however, higher rather than lower socioeconomic status was associated with significantly higher risk of TB infection. Our study confirmed this conclusion, suggesting that TB transmission in our study sites may occur through exposure to as yet unidentified risk factors associated with higher socioeconomic status.

Our study had some limitations. First, as a longitudinal study, one-year follow-up is too short to obtain more useful effects. Second, our study did not include some interesting key variables that have been studied in other settings such as HIV infection, effect of drug resistant patterns and use of mass public transportation due to restriction of conditions, so there might be selection bias. These variables will be included in the further research of our study to examine relative risk factors of TB.

### Conclusions

TB incidence in rural populations is very high in China. Considering the large agricultural population and characteristics of this disease, TB is still a severe public health challenge in China and may need effective strategies to decrease the TB burden in rural areas.
